# Neodymium-140 DOTA-LM3: Evaluation of an *In Vivo* Generator for PET with a Non-Internalizing Vector

**DOI:** 10.3389/fmed.2017.00098

**Published:** 2017-07-12

**Authors:** Gregory W. Severin, Lotte K. Kristensen, Carsten H. Nielsen, Jesper Fonslet, Andreas I. Jensen, Anders F. Frellsen, K. M. Jensen, Dennis R. Elema, Helmut Maecke, Andreas Kjær, Karl Johnston, Ulli Köster

**Affiliations:** ^1^Hevesy Laboratory, DTU Nutech, Technical University of Denmark, Roskilde, Denmark; ^2^Department of Chemistry, Michigan State University, East Lansing, MI, United States; ^3^Facility for Rare Isotope Beams, Michigan State University, East Lansing, MI, United States; ^4^Department of Clinical Physiology, Nuclear Medicine & PET and Cluster for Molecular Imaging, Rigshospitalet, University of Copenhagen, Copenhagen, Denmark; ^5^Department of Nuclear Medicine, University Hospital Freiburg, Freiburg, Germany; ^6^ISOLDE, CERN, Geneva, Switzerland; ^7^Institut Laue-Langevin, Grenoble, France

**Keywords:** *in vivo* generator, ^140^Nd, ^140^Pr, internalization, positron emission tomography, DOTA-LM3

## Abstract

^140^Nd (*t*_1/2_ = 3.4 days), owing to its short-lived positron emitting daughter ^140^Pr (*t*_1/2_ = 3.4 min), has promise as an *in vivo* generator for positron emission tomography (PET). However, the electron capture decay of ^140^Nd is chemically disruptive to macrocycle-based radiolabeling, meaning that an *in vivo* redistribution of the daughter ^140^Pr is expected before positron emission. The purpose of this study was to determine how the delayed positron from the de-labeled ^140^Pr affects preclinical imaging with ^140^Nd. To explore the effect, ^140^Nd was produced at CERN-ISOLDE, reacted with the somatostatin analogue, DOTA-LM3 (1,4,7,10- tetraazacyclododecane, 1,4,7- tri acetic acid, 10- acetamide N - p-Cl-Phecyclo(d-Cys-Tyr-d-4-amino-Phe(carbamoyl)-Lys-Thr-Cys)d-Tyr-NH2) and injected into H727 xenograft bearing mice. Comparative pre- and post-mortem PET imaging at 16 h postinjection was used to quantify the *in vivo* redistribution of ^140^Pr following ^140^Nd decay. The somatostatin receptor-positive pancreas exhibited the highest tissue accumulation of ^140^Nd-DOTA-LM3 (13% ID/g at 16 h) coupled with the largest observed redistribution rate, where 56 ± 7% (*n* = 4, mean ± SD) of the *in situ* produced ^140^Pr washed out of the pancreas before decay. Contrastingly, the liver, spleen, and lungs acted as strong sink organs for free ^140^Pr^3+^. Based upon these results, we conclude that ^140^Nd imaging with a non-internalizing vector convolutes the biodistribution of the tracer with the accumulation pattern of free ^140^Pr. This redistribution phenomenon may show promise as a probe of the cellular interaction with the vector, such as in determining tissue dependent internalization behavior.

## Introduction

**Figure 1:**
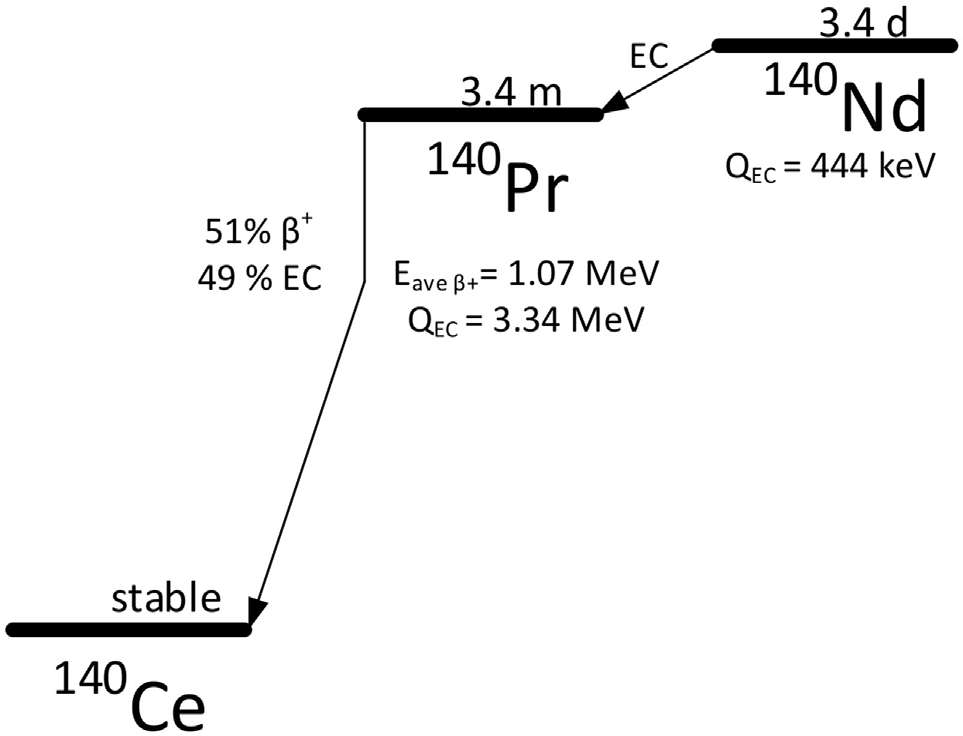
The decay scheme of ^140^Nd and its daughter ^140^Pr.

The demand for long-lived positron emitting radiolanthanides is growing due to the success of targeted internal radiotherapy with ^177^Lu, and the promise of other therapeutic lanthanides such as Auger electron emitters ^165^Er, and ^135^La or combined beta-/Auger electron emitters such as ^161^Tb (1–5). Neodymium-140 (^140^Nd, *t*_1/2_ = 3.4 days) decays to praseodymium-140 (^140^Pr, *t*_1/2_ = 3.4 min) by electron capture with no emission of gamma photons (Figure 1) (6). Because ^140^Pr has a 51% positron branch (*E*_mean_ = 1.07 MeV) and a short half-life, the pair has potential for long-lived positron emission tomography (PET) tracing of pharmaceuticals. Together as a so-called *in vivo generator* (7, 8), they provide a high positron yield with lanthanide labeling chemistry and a parent half-life that is suitable for monoclonal antibody, nanoparticle, and peptide imaging (9, 10). In this light, it is interesting to pursue development of ^140^Nd to investigate how the delayed positron emission from ^140^Pr affects medical imaging, and how it can be exploited.

The radionuclide ^140^Nd is non-standard in radiopharmacy, but can be produced in a variety of methods: *via* (*p,2n*) reactions on naturally monoisotopic praseodymium-141 (11), ^3^He bombardment of natural cerium (11–13), or by spallation processes on tantalum (14). Due to the relatively lower volatilization temperature of the rare earths (compared to tantalum), it is possible to extract the spallation-induced radiolanthanides by thermal diffusion and separate them by mass, e.g., at the on-line separator ISOLDE at CERN (15, 16). In principle, this leads to the highest possible specific activity for radiochemistry and has been successfully employed in previous experiments with radiolanthanides (3, 17, 18). The (p,2n) production method is attractive for future developments because it is a reaction that is reachable by biomedical and hospital cyclotrons. However, it requires very high purity praseodymium starting material (without neodymium contamination), and a robust lanthanide separation technique in order to achieve radiolabeling with high specific activity.

When considering ^140^Nd for PET, its value for direct imaging hinges upon the chemical and kinetic profile of the positron producing daughter nuclide ^140^Pr. Previous reports show that the EC decay of DOTA-bound ^140^Nd is highly efficient at releasing the daughter ^140^Pr from the chelate, making it available for further interactions as a Pr^3+^ cation (13). Praseodymium and neodymium have remarkably similar chemistry, and under the right conditions Pr^3+^ could be predicted to re-bind a free chelator after being released. However, an activation barrier to stable binding between lanthanide ions and DOTA precludes room temperature chelation of the Pr^3+^ daughter. Other chelators such as DTPA are not inhibited by an activation barrier and may fare better at retaining the daughter praseodymium. The mobility of the daughter praseodymium determines how different the distribution of the tracer-bound parent is from the distribution of the unbound daughter, the evaluation of which will determine the value of ^140^Nd/^140^Pr with functionalized DOTA *in vivo*. The current set of experiments serves as a preliminary investigation into the ^140^Nd/^140^Pr *in vivo* PET generator.

In order to create the appropriate scenario to test ^140^Nd/^140^Pr, the somatostatin receptor was selected as the target. The reason for this is threefold. First, somatostatin analogs are already employed clinically with the therapeutic radionuclide ^177^Lu (19) and the diagnostic radionuclides ^64^Cu (20) and ^68^Ga, where a long-lived positron emitting lanthanide could prove useful in dose determinations. Second, the depth of research into somatostatin receptors has led to the development of well-established internalizing vectors (21), such as DOTATATE, and non-internalizing vectors (22) such as DOTA-LM3 (23, 24). And third, the receptor is expressed in the pancreas, but not in many other tissues: thereby providing a test-tissue with more realistic perfusion than xenograft tumors. In the present study, testing was performed with DOTA-LM3 anticipating that the redistribution of praseodymium would be most evident with a targeting vector that remained located on the surface of the targeted cells.

Herein, we present the results from ^140^Nd-DOTA-LM3 PET quantifications in H727 xenograft tumor-bearing mice before and after euthanasia. The pre- and post-mortem images represent the daughter and parent radionuclide distributions, respectively. As positrons are only emitted by the daughter, PET scanning only reveals the parent distribution in the absence of biological processes that differentiate the vector bound parent from the daughter. We also show verification of the dislocation (also referred to as de-labeling) of ^140^Pr from DOTA-LM3 by radio-HPLC. Furthermore, *ex vivo* biodistributions from ^140^Nd-DOTA-LM3 and ^140^Nd as the free ion are used to show the source and sink organs for the free praseodymium daughter.

## Materials and Methods

### General

All water was 18 MΩ-cm MilliQ purified and was used to produce all aqueous solutions. Hydrochloric acid solutions were prepared from concentrated HCl (TraceSelect, Sigma).

### Production of ^140^Nd

A 55 g/cm^2^ tantalum foil target was irradiated by a 1.4 GeV proton beam, creating a multitude of radioactive and stable spallation products. The product nuclei, lanthanides in particular, diffused from the ≈2,000°C target to a ≈2,000°C tungsten surface ionizer. The ions were extracted at 30 kV and mass-separated with a 70° sector magnet (1.5 m mean bending radius). The *A* = 140 beam was implanted into two Zn-coated gold foils. The zinc, totaling 2–3 mg had been electrodeposited onto the gold foils over approximately 0.5 cm^2^. The entire procedure is nearly identical to the methodology recently described for projects collecting Tb isotopes at ISOLDE (3, 17).

### Radiochemistry

The following methodology was carried out two times with small variations between each run.

The zinc layer containing ^140^Nd was etched briefly with aq. HCl (200 µL, 2 M), and the resulting solution was diluted to 2.2 mL with aq. HCl (2 M). This was heated to 98°C, cooled, and passed over AG1x8 anion exchange resin (300 mg, Biorad, 200–400 mesh, initially formate-form) packed in a 4 mm internal diameter (ID), fritted polypropylene column (Supelco) that had been prepped by three times sequential washing with water (3× bed volume), 2 M HCl (3× bed volume), and 6 M HCl (3× bed volume), finishing with equilibration in 2 M HCl. The resin has a high affinity for [ZnCl_4_]^2−^ ions in 2 M HCl (25) and was intended to remove any zinc impurity from the ^140^Nd. An additional 0.5 mL of 2 M HCl was used to rinse residual ^140^Nd from the column, and the entire effluent was collected and adjusted to pH 5–6 with aq. ammonium acetate (1 M, pH 7) and aq. ammonium hydroxide (28%, TraceSelect, Sigma) to a final acetate concentration of roughly 200 mM. This solution was passed over a hydroxamate functionalized Waters CM resin bed (26) (100 mg, 4 mm ID) to trap the ^140^Nd and was washed with water (7 mL), then eluted with aq. HCl (600 µL, 0.1 M). The eluent was kept as the stock ^140^Nd solution for further radiolabeling and formulation.

For the DOTA-LM3 preparation, 220 µL of the ^140^Nd stock solution was added to aq. ammonium acetate (780 µL, 300 mM). Two productions were made, the first with 9 µg DOTA-LM3 (added from a stock solution of 1 mg/mL in water) and the second with 18 µg DOTA-LM3. The solution was heated in a sealed container to 95°C and incubated for 25 min. After cooling to room temperature, the labeling reaction was quenched with aq. DTPA (12 µL, 0.5 mM, pH 7) and let stand for 5 min. The solution was passed over a Waters C18 light sep-pak (prepped with 10 mL ethanol and 10 mL water) to trap the labeled product, rinsed with 1 mL water, and then eluted with 2 mL ethanol. The ethanol solution was taken almost to dryness (residual volume was approximately 50 µL) with the residual being diluted with 900 µL HEPES buffered isotonic saline (10 mM HEPES, 150 mM NaCl, pH 7.4) and was used directly for injections.

The neodymium “chloride” injections were prepared by diluting 100 µL of ^140^Nd stock (in 0.1 M HCl) with 390 µL HEPES buffered isotonic saline (10 mM HEPES), and neutralizing with 10 µL 1 M Na-HEPES.

### HPLC Verification of De-Labeling and RadioTLC for Determination of Radiochemical Purity

Samples of ^140^Nd-DOTA-LM3 were injected onto a reverse-phase C-18 (Luna 3uC18(2)(n) 100 A 100 × 2 mm 3 μm, Phenomenex) column at a flow rate of 0.5 mL/min starting from 0% acetonitrile in water, and reaching 100% over a 15 min gradient. Elution was monitored with a radio detector. The entire effluent was collected in 1 min intervals (500 µL each) and quantified 4 days after collection by liquid scintillation counting on a HIDEX 300 SL spectrometer.

RadioTLC was performed by spotting 1 µL of the DOTA-LM3 solutions (before and after C-18 purification) onto aluminum-backed silica TLC sheets. The sheets were eluted in 10% (w/v) *aq*. CH_3_COONa:CH_3_OH (1:1). Unreacted ^140^Nd remained at the origin, and ^140^Nd-DOTA-LM3 moved to R_f_ ~0.5.

### PET Imaging and *Ex Vivo* Biodistributions

NCI-H727 lung carcinoid cancer cells (ATCC CRL-5815, LGC Standards) were cultured in RPMI-1640 media supplemented with 10% fetal bovine serum and 1% penicillin-streptomycin (Invitrogen) at 37° C and 5% CO_2_. Cells in their exponential growth phase and at 80–90% confluence were harvested by trypsinization and resuspended in 1:1 media and matrigel (BD Biosciences) at 5 × 10^7^ cells/mL. Subcutaneous tumors were established in female NMRI nude mice (Taconic, Denmark) by inoculation of 5 × 10^6^ cells in 100 µL on each flank above the hind limbs in the subcutaneous space. All animal experiments were performed under a protocol approved by the National Animal Experiments Inspectorate of Denmark.

Longitudinal small animal PET/CT imaging (Inveon Multimodality PET/CT scanner, Siemens) was performed with NCI-H727 tumor bearing mice injected intravenously with 3.3–4.3 MBq ^140^Nd-DOTA-LM3 (*n* = 8) or 2.7–3.1 MBq ^140^Nd-chloride (*n* = 3) in 150 µL. Mice were anesthetized with sevoflurane (Abbott Laboratories) during injection and PET/CT imaging. PET data was acquired for 600 s in list mode at 1, 3, and 16 h after injection. The mice were sacrificed after the 16 h time-point and a PET acquisition was performed 2 h post-mortem. Images were reconstructed using a 3D maximum *a posteriori* algorithm with CT based attenuation correction. CT images were acquired with the following settings: 300 projections, 65 kV, 500 µA, and 400 ms exposure, and reconstructed with an isotropic voxel size of 105 µm. Image analysis was performed using the Inveon Software (Siemens). Region of interests (ROIs) were drawn manually over the tumor regions and other organs based on the CT images and the uptake of ^140^Nd-DOTA-LM3 or ^140^Nd-chloride quantified as % injected dose per gram tissue (%ID/g).

Conventional *ex vivo* biodistribution was performed after the post-mortem scan. Tumors and organs were resected, weighted and the radioactivity was counted in a gamma counter (Wizard^2^, PerkinElmer).

## Results and Discussion

### Isolation of ^140^Nd at ISOLDE, Radiochemical Purification, and Radiolabeling of ^140^Nd-DOTA-LM3

^140^Nd was produced by 1.4 GeV proton induced spallation of tantalum at ISOLDE. The process for vaporization and ionization of lanthanides at the ISOLDE facility is well described (15), and proceeded without complication. The electromagnetic separation of proton rich *A* = 140 spallation products led to a total of about 530 MBq (in two productions) of >99% radionuclidic purity ^140^Nd in two Zn-coated gold foils. The ^140^Nd implanted foils were briefly etched (without fully dissolving the entire Zn layer) with aq. hydrochloric acid (HCl, 2 M) and the carrier Zn (approximately 1 mg Zn^2+^ in 2 mL 2 M HCl) was removed by passage over AG1x8 anion exchange resin. ICP-OES measurement showed that Zn was completely adsorbed onto the resin, with <30 ng Zn remaining in the purified ^140^Nd stock solution (~60 MBq). ^140^Nd was concentrated by trap-and-release on a small mixed-bed hydroxamate/carboxylate-functionalized resin. Trapping was only efficient after heating the solution for several minutes at 95°C, indicating that the ^140^Nd may not have been completely dissolved during the initial Zn etching. When the 2 M HCl etch solutions containing the Zn were heated prior to purification, the trapping on the hydroxamate/carboxylate resin exceeded 99% efficiency. The release of ^140^Nd from the resin was accomplished by elution with aq. HCl (600 µL, 0.1 M) at 98% efficiency.

The eluted ^140^Nd was reacted with DOTA-LM3 in ammonium acetate buffer (300 mM, pH 4.8), and after 30–60 min at 95°C radioTLC indicated that ^140^Nd-DOTA-LM3 had formed in a 75% radiochemical yield. Quenching with DTPA and C-18 sep-pak purification led to an ultimate combined radiochemical yield and recovery of 60% in HEPES-buffered saline (pH 7.4). RadioTLC after C-18 purification indicated >95% radiochemical purity of ^140^Nd-DOTA-LM3. The production, purification, and labeling procedure was performed twice, where the amount of peptide relative to radioactivity was selected based upon titration. Although it would have been ideal to have all samples at identical specific activity, it was not possible, and in this case, the final radiolabeled specific activities for ^140^Nd-DOTA-LM3 were 5.0 and 2.5 MBq/nmol, each with sufficient activity for injection of four mice (see below).

^140^Nd in an unchelated form was prepared for injection by pH adjustment of the eluted ^140^Nd stock solution with sodium HEPES and diluted to a final formulation in pH 7.4, 150 mM NaCl, and 10 mM HEPES. Characterization of the ^140^Nd chemical species was not performed, and it is herein referred to as “^140^Nd-chloride” for convenience.

### HPLC-Traces of ^140^Nd DOTA-LM3

**Figure 2:**
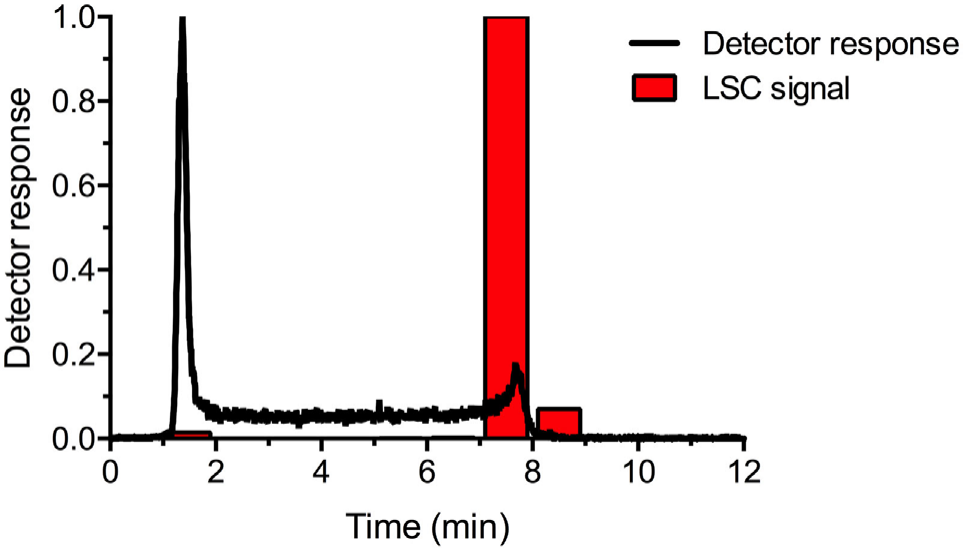
Effluent analysis of ^140^Nd-DOTA-LM3 in equilibrium with the daughter, ^140^Pr, from reverse-phase HPLC. The black line depicts the immediate radiotrace, showing the distribution of the daughter, ^140^Pr. The red bars give the LSC signal from collected fractions, 1 min each, analyzed >4 days after elution, illustrating the parent, ^140^Nd, distribution. Detector response is relative and scaled to the maximum for each trace.

Purified ^140^Nd-DOTA-LM3 was analyzed on reverse-phase HPLC to highlight the parent-daughter dechelation effect. The relative quantifications of the HPLC effluent are shown overlaid in Figure 2. The product ^140^Nd-DOTA-LM3 eluted at 7.9 min, which agreed with the equilibrated liquid scintillation counter (LSC) trace, confirming >95% radiochemical purity of the ^140^Nd-DOTA-LM3. It should be noted that parent-daughter transient equilibrium was reached before the LSC samples were counted, meaning that the LSC signal was representative of the parent ^140^Nd elution profile. In contrast, the online radio-detector trace demonstrated the daughter ^140^Pr behavior. This is because the detector was more sensitive to gamma radiation arising from positron annihilation after ^140^Pr decay, and because of the relatively low abundance of penetrating radiation arising from ^140^Nd decay. Here, ^140^Pr was shown to elute with the solvent front with an elevated baseline between the solvent peak and elution of the parent ^140^Nd-DOTA-LM3. Since the ^140^Pr^3+^ eluted faster than the radiolabeled peptide, the solvent front peak was evidence of the formation of ^140^Pr^3+^ in the injected solution, and the elevated baseline showed the *in situ* formation and washout of ^140^Pr^3+^ from ^140^Nd decaying on the column. From the trace, it was evident that the release of ^140^Pr from DOTA-LM3 after ^140^Nd decay is >95% efficient, matching the observations of Zhernosekov and co-workers (13). Furthermore, the column behavior illustrated the expected *in vivo* behavior: a rapid redistribution of the positron-emitting daughter after the decay of the parent.

### *Ex Vivo* Biodistributions of ^140^Nd-DOTA-LM3 and ^140^Nd-Chloride

Eight mice bearing dual-flank NCI-H727 lung carcinoid tumors were injected with 3–4 MBq ^140^Nd-DOTA-LM3: four at a specific activity of 5 MBq/nmol and four at 2.5 MBq/nmol. A further three tumor-bearing mice were injected with 3 MBq ^140^Nd-chloride. PET quantifications were obtained at 1, 3, and 16 h postinjection. After the last scan the animals were euthanized, and following equilibration of the daughter, the mice were rescanned. Finally, the animals were dissected, and tissue samples were weighed and counted.

**Figure 3:**
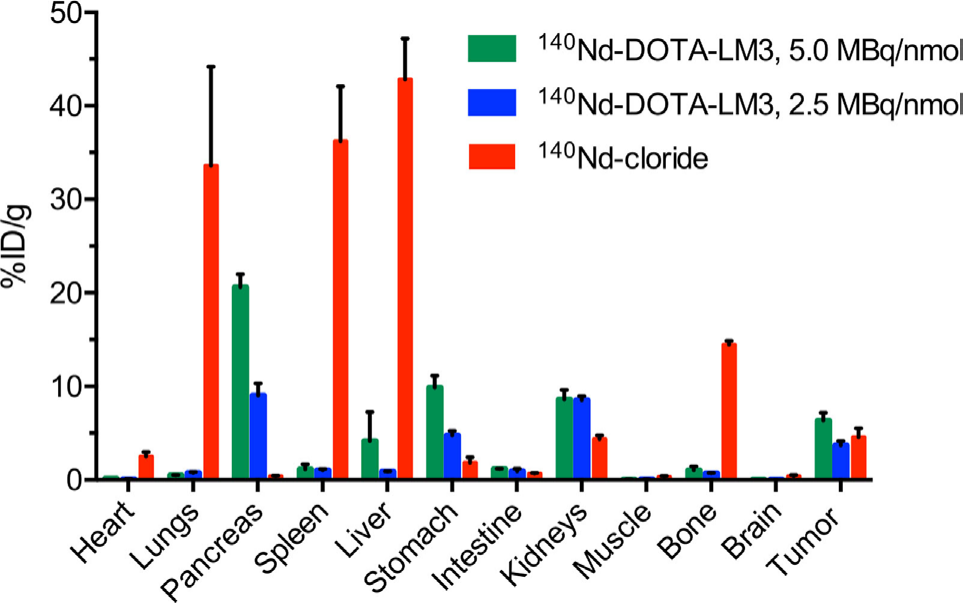
*Ex vivo* biodistribution of ^140^Nd-DOTA-LM3 and ^140^Nd-chloride. Animals were euthanized 16 h after injection of 3.3–4.3 MBq ^140^Nd-DOTA-LM3 (5.0 MBq/nmol, 0.5 nmol injected, *n* = 4), ^140^Nd-DOTA-LM3 (2.5 MBq/nmol, 1 nmol injected, *n* = 4) and 2.7–3.1 MBq ^140^Nd-chloride (*n* = 3). Organ counting commenced after parent/daughter equilibrium was achieved (2 h after euthanasia). ^140^Nd-DOTA-LM3 quantifications give the “source” distribution for ^140^Pr in the 16 h positron emission tomography studies, and ^140^Nd-chloride qualitatively describes the “sink” behavior. Data are depicted as mean ± SEM.

For the unbound ^140^Nd-chloride injections, the *ex vivo* biodistribution (16 h) showed high levels of accumulation in the lungs, spleen and liver, and to a lesser extent bone and tumor (Figure 3). This is a typical biodistribution for free +3 oxidation state radiolanthanides [for femur and liver vs. tumor, see, Ref. (27)] and for other hard radiometals [e.g., see, Ref. (24)]. The tissues with high accumulation of ^140^Nd were also expected to accumulate released ^140^Pr, owing to the chemical similarities between praseodymium and neodymium. Thus, this biodistribution serves as a descriptor of which tissues we expect to exhibit “sink” behavior in the pre-mortem PET scans.

The *ex vivo* biodistribution of ^140^Nd-DOTA-LM3 was very distinct from that of ^140^Nd-chloride with the pancreas, a known somatostatin receptor positive organ, being particularly interesting (21). The pancreas was found to take up ^140^Nd-DOTA-LM3 without accumulating the free radiometal (13% ID/g with high specific activity ^140^Nd-DOTA-LM3, compared to 0.1% ID/g with the free ^140^Nd^3+^). Therefore, in the PET study, the pancreas was expected to have a lower signal in the pre-mortem ^140^Nd-DOTA-LM3 scans compared to the post-mortem scans. The liver, spleen, and lungs had the opposite behavior, accumulating the free radiometal but not the peptide, and were expected to have a higher signal in the pre-mortem scans than in the post-mortem.

Fani et al. showed that in comparing the biodistribution of ^68^Ga-NODAGA-LM3, ^68^Ga-DOTA-LM3, ^64^Cu-NODAGA-LM3, and ^64^Cu-CBTE2A-LM3, tumor uptake relative to the pancreas, stomach, and kidney is highly variable depending on the metal and chelator used (23, 28). In the current work, we injected 300 pmol (5 MBq/nmol) or 600 pmol (2.5 MBq/nmol) in order to get enough signal for the PET scans. For the biodistributions from Fani et al., 10 pmol was injected, and blocking was performed with 200 nmol of excess DOTA-LM3. The overall effect of the blocking was to reduce the uptake in sst2 expressing tissues, which is exactly what was observed between the higher and lower specific activity injections of the current study: a modest reduction in the uptake in sst2 expressing tissues relative to the kidney, and overall faster excretion.

Based upon the *ex vivo* biodistribution, the tumors had an intermediate behavior, weakly concentrating both the free ^140^Nd and the labeled peptide, meaning that the PET signal was expected to change little between the pre- and post-mortem scans (i.e., washout of ^140^Pr from the tumor volume could be compensated by uptake of ^140^Pr from the bloodstream). This behavior is in many ways an undesirable outcome, because signals arising from the tumors can be attributable to both the free metal ions and the targeted peptide. Future work with a different vector/target system could give a result with a simpler interpretation. Nevertheless, the pancreas remains an interesting tissue within the present set of experiments.

### PET Studies Show Tissue Dependent Redistribution of ^140^Pr after ^140^Nd Decay

**Figure 4:**
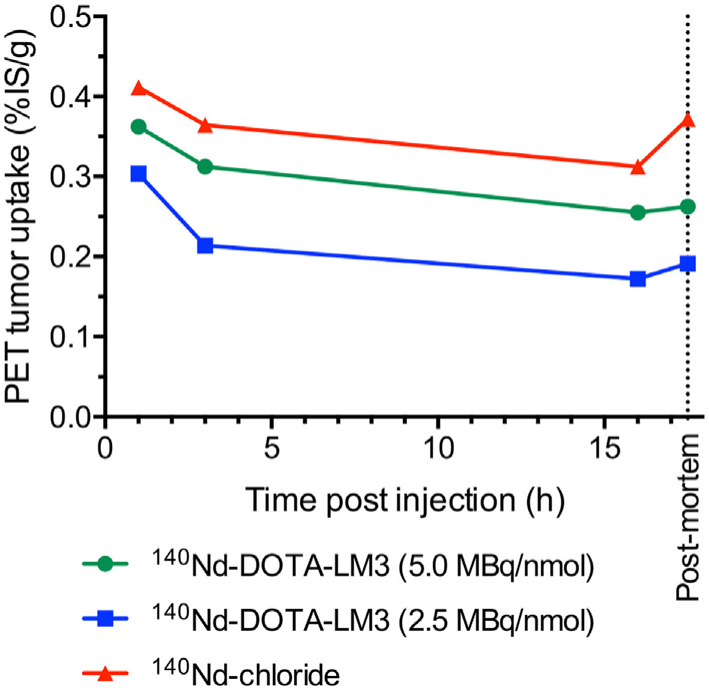
Positron emission tomography (PET) signal in tumors as a function of time after injection with 3.3–4.3 MBq of ^140^Nd-DOTA-LM3 or 2.7–3.1 MBq of ^140^Nd-chloride. Values are presented as %ID/g and depicted as mean ± SEM (^140^Nd-DOTA-LM3, *n* = 8 each and ^140^Nd-cloride, *n* = 6). Animals were anesthetized with sevoflurane during the 600 s PET acquisitions.

**Figure 5:**
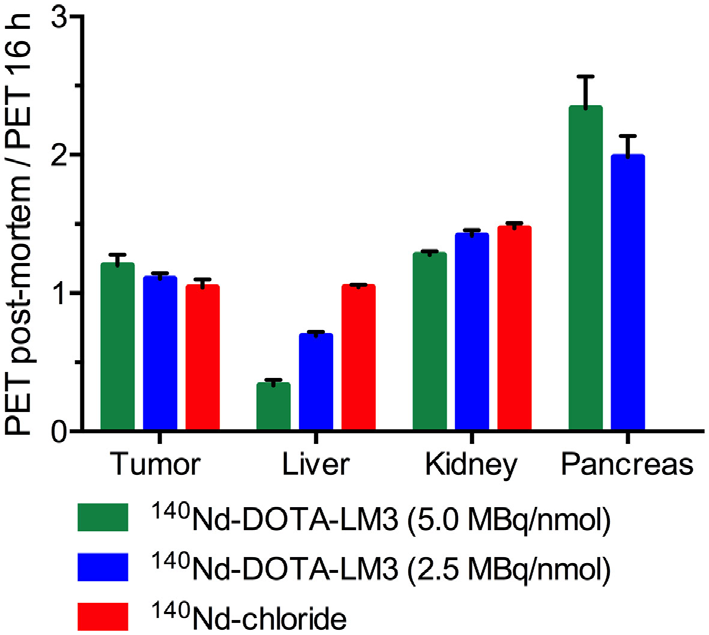
Ratio of the post-mortem positron emission tomography (PET) signal to the pre-mortem (16 h) PET signal for each tissue. Tissues with ratios greater than unity release ^140^Pr into circulation faster than they absorb it. ^140^Pr is trafficked away from the site of ^140^Nd decay. The pancreatic signal from the ^140^Nd-chloride images was masked by the high liver signal, prohibiting reliable quantification.

Positron emission tomography data were analyzed to quantify the ^140^Pr signal from the tumors, pancreas, kidney, lung, and liver (Table 1). The PET signals for the tumors are given as the percent of the injected signal per gram (%IS/g) (Figure 4). This unit is non-standard and is described further in the Supplementary Material. %IS/g was chosen for two reasons: first because the PET scans quantify the redistributed daughter ^140^Pr, not the ^140^Nd-tracer, and second because the data were not corrected for point spreading due to extensive positron range. This means that there is a substantial partial volume distortion when converting from annihilation signal (%IS/g) to tracer concentration (%ID/g), and that the well-counter based *ex vivo* biodistribution is not directly comparable to the post mortem PET results. A discussion of the partial volume effect due to the high energy positrons from ^140^Pr is included in the Supplementary Material. The tumor time-activity curve shows that in this model (as expected from the *ex vivo* biodistribution) very little redistribution is observed. For the present case, the tumor was known to accumulate the trivalent lanthanide to some small degree (from the Nd-chloride injection data above), meaning that ^140^Pr^3+^ produced by decay in the tumor region had some tendency to remain. In other organs, however, due to the dechelation effect, there was a dramatic change in the PET signal in the pre- and post-mortem imaging quantifications. The redistribution effects are displayed in Figure 5 as the ratio of the mean organ signal between the post-mortem, and pre-mortem (16 h) images. This effect is most evident in the pancreas and liver for the high specific activity injections, where the decrease in liver signal is matched by an increase in the pancreas signal, confirming the behaviors of the peptide and free metal observed in the *ex vivo* biodistribution.

**Table 1:** Positron emission tomography tissue quantifications and paired-difference two-tailed *t*-test *p*-values.

Subject#	L. tumor		R. tumor			Muscle			Kidney			Liver			Pancreas			Lung		
	16 h	PM	16 h	PM	*p*	16 h	PM	*p*	16 h	PM	*p*	16 h	PM	*p*	16 h	PM	*p*	16 h	PM	*p*
**5 MBq/nmol DOTA-LM3**																				
1	0.28	0.38	0.23	0.29	**1.9E−2**	0.05	0.08	5.7E**−**1	1.09	1.41	**2.7E−3**	0.53	0.21	**2.2E−4**	0.85	1.79	**1.2E−3**	0.15	0.06	**2.4E−3**
2	0.24	0.35	0.29	0.41		0.06	0.07		0.94	1.26		0.53	0.17		0.71	2.12		0.16	0.08	
3	0.59	0.68	0.44	0.44		0.06	0.06		1.41	1.76		0.62	0.25		1.29	2.56		0.19	0.08	
4	0.23	0.24	0.20	0.18		0.08	0.06		0.94	1.15		0.53	0.12		1.00	2.26		0.17	0.04	
**2.5 MBq/nmol DOTA-LM3**																				
5	0.26	0.29	0.25	0.28	**1.1E−2**	0.05	0.04	2.4E**−**1	0.74	1.12	**2.4E−3**	0.29	0.20	**6.5E−4**	0.53	0.76	**1.2E−3**	0.15	0.06	**2.4E−3**
6	0.15	0.17	0.08	0.07		0.04	0.03		0.94	1.35		0.29	0.23		0.79	1.71		0.16	0.08	
7	0.16	0.18	0.18	0.19		0.02	0.03		0.71	0.97		0.25	0.17		0.41	0.88		0.19	0.08	
8	0.18	0.18	0.14	0.18		0.03	0.03		0.88	1.18		0.24	0.15		0.41	0.82		0.17	0.04	
**^140^Nd-chloride**																				
9	0.32	0.29	0.32	0.32	6.0E**−**1	0.22	0.23	1.2E**−**1	0.79	1.12	**1.1E−2**	10.50	10.85	1.5E**−**1	N/Q			1.71	1.62	6.1E−1
10	0.23	0.24	0.22	0.21		0.19	0.23		0.88	1.35		10.73	11.00					2.35	2.32	
11	0.22	0.22	0.22	0.29		0.24	0.28		0.88	1.29		12.50	13.49					3.09	3.47	

*The tissue signal quantifications in %IS/g are given for the 16 h postinjection scans (16 h) and the post mortem scans (PM). The paired-difference *t*-test was used to indicate in which cases the pre- and post-mortem quantifications were significantly different, where *p*-values under 0.05 are displayed in bold. Each subject had two tumors, and the quantification for each is used for the computation of the tumor *p*-value. The high concentration of the ^140^Nd in the liver of subjects receiving the ^140^Nd-chloride injections precluded quantification of activity in the pancreas (N/Q, not quantified)*.

It should be noted that the injected mass of the tracer had a large effect upon the PET quantifications and *ex vivo* biodistribution (Figures 4 and 5). This indicates that the receptor-specific accumulation was becoming saturated as the injected mass increased from 0.5 nmol (5.0 MBq/nmol) to 1 nmol (2.5 MBq/nmol). This saturation behavior is non-desirable for probing the internalization behavior of the peptide as non-specific interactions begin to dominate the tracer distribution.

**Figure 6:**
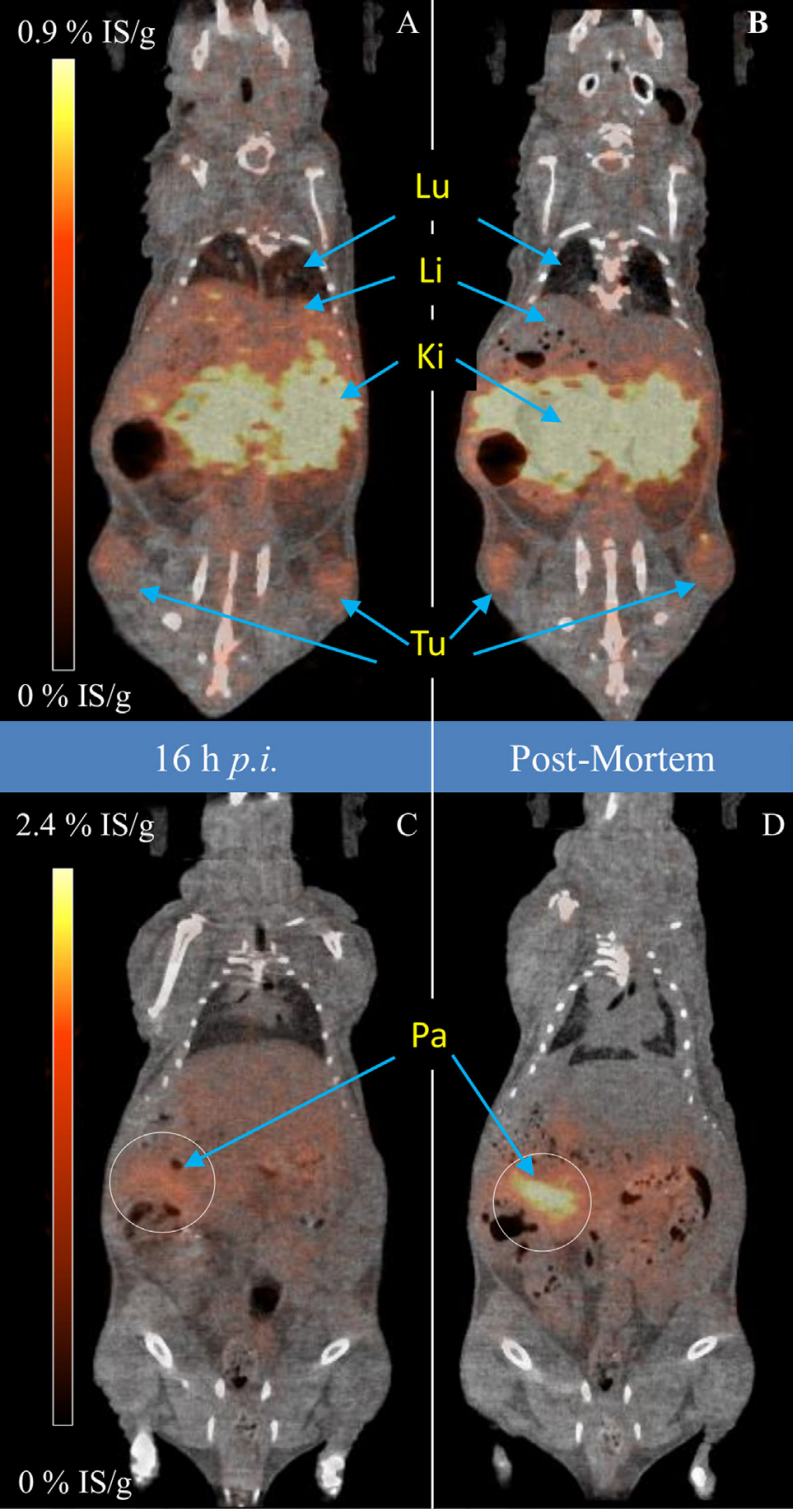
Pre-mortem [left **(A,C)**] and post-mortem [right **(B,D)**] positron emission tomography/CT scan of a mouse 16 h after injection with 2.9 MBq ^140^Nd-DOTA-LM3 (5.0 MBq/nmol). The upper frames are shown with a different scale in order to allow visualization of the sink behavior of the liver and lungs. In the lower frames, a white circle is drawn over the pancreas (somatostatin receptor expressing) where the difference in the pre- and post-mortem pancreatic signal is due to rapid diffusion of ^140^Pr from the highly perfused pancreas into the blood stream. Data were acquired over 600 s. Pre-mortem anesthesia was sevoflurane (Lu, lungs; Li, liver; Ki, kidney; Tu, tumor; Pa, pancreas).

Figure 6 shows an example PET/CT reconstruction, qualitatively illustrating the redistribution effect. The most striking differences in the images between the daughter distribution (16 h, left panels) and the parent distribution (post-mortem, right panels) are seen in the liver, lungs, and pancreas. As expected, the 16 h pre-mortem PET images more closely reflected the biodistribution observed for the free ^140^Nd-chloride injections, while the post-mortem images showed the biodistribution for the intact ^140^Nd-DOTA-LM3 tracer.

The most important result from the PET imaging was the pancreatic signal. In this case, in the pre-mortem image, it was difficult to delineate the pancreas due to the elevated-background in the liver. This is despite the fact that the *ex vivo* biodistribution revealed that the pancreas contained 13%ID/g of the tracer: which should be easily distinguishable from the 3%ID/g of the liver. The discrepancy is resolved in the post-mortem imaging, where the accumulation of the tracer in the pancreas, and not in the liver, is apparent. The effects observed are consistent with a hypothesis that ^140^Nd-DOTA-LM3 localized on the surface of pancreatic cells (via non-internalizing interactions with the somatostatin receptor) releases ^140^Pr^3+^ into the blood stream, where it is quickly redistributed to the liver, spleen and lungs. In fact, results from the higher specific activity ^140^Nd-DOTA-LM3 studies show that 56 ± 7% (*n* = 4, mean ± SD) of the *in situ* produced ^140^Pr washed out of the pancreas before decay.

For statistical analysis the pre- and post-mortem PET quantifications were compared with a paired-difference two-tailed *t*-test. The tabulated data are presented in Table 1, along with the *p* values. In this case, the *t*-test was used to determine the significance of the *absolute difference* between the pre- and post-mortem signals in %IS/g. Admittedly, there are many ways to analyze these data, and in this case, the paired-difference test was selected because it adds to the statistical power by comparing the tissues in a single subject to themselves after intervention. Clearly from the *p* values in Table 1, the use of DOTA-LM3 as a tracer led to significant changes from the pre- to post-mortem images, while the non-targeted ^140^Nd-chloride remained largely unaltered. Of note in the free ion ^140^Nd-chloride injections, is that the only tissue with a statistically significant difference between the pre- and post-mortem quantification is the kidney, whereas in the targeted DOTA-LM3 images the only tissue lacking a significant difference was the muscle. The kidney is interesting because in all cases the post-mortem signal was higher, by 25–50% than the pre-mortem value. While it could be suggested that this is due to rapid excretion of ^140^Pr^3+^ from the kidney to the bladder *in vivo*, the fact that this is also observed with the ^140^Nd-chloride injections indicates that within the kidney the chemical form of the neodymium is not necessarily as a free cationic lanthanide. However, in all other tissues, the distribution of ^140^Nd from the ^140^Nd-chloride injections strongly resembles that of redistributed ^140^Pr.

**Figure 7:**
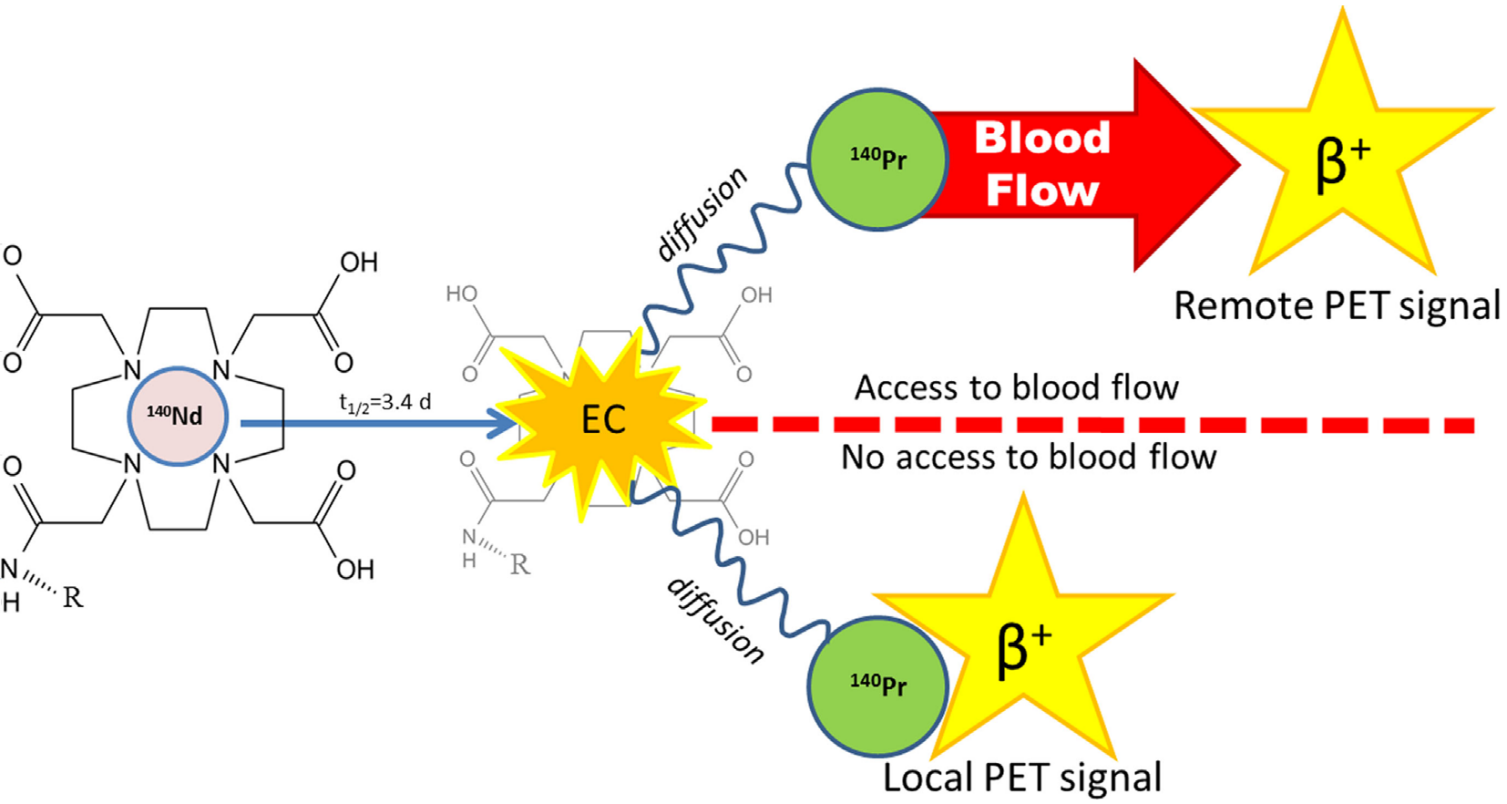
A conceptual drawing of the experimental hypothesis. A tracer (R), is labeled by DOTA-bound ^140^Nd which decays by electron capture (EC) to ^140^Pr. The resulting atomic rearrangement releases the daughter ^140^Pr from DOTA as a free ion. If ^140^Pr diffuses into the blood stream before decaying (upper path), it will be carried away and give a remote positron emission tomography (PET) signal. However, if there is a diffusion barrier such as a cell membrane or a lack of blood flow (lower path), the PET signal will remain localized to the tracer accumulation site.

The pancreatic washout reveals a potential benefit derived from the ^140^Pr’s delayed positron. Specifically, the degree of redistribution of ^140^Pr^3+^ may be affected by its location and access to blood flow. This means that the PET signal observed with ^140^Nd labeled vectors might be highly dependent on their cellular internalization status, as ^140^Pr^3+^ cations originating from decays occurring on the surface of the cell or in circulation may be transported away by the blood flow, whereas^140^ Pr^3+^ cations released from ^140^Nd decay inside of a cell have an additional diffusion barrier. A conceptual graphic demonstrating the idea is depicted in Figure 7. As many promising new classes of pharmaceuticals, in particular nanoparticle drug formulations, gene therapy and targeted Auger emitting radionuclides, are expected to be most effective when internalized, a PET radiolabel for determining internalization would be a valuable tool for drug development.

### General Discussion

When searching for long-lived PET radionuclides, the utility of ^140^Nd is immediately evident in its half-life and lack of concurrent gamma emissions. However, due to the nature of the delayed positron from the short-lived daughter, it is important to understand how imaging may be affected by dechelation. The redistribution of ^140^Pr in the present case was clearly visible in the pre- and post-mortem PET images. While the tumor signal was significantly changed, the magnitude of change was small which may preclude application. However, this behavior might be model dependent, and not general for all tumor types or locations. Nevertheless, the signals from the other tissues show the potential for using the daughter-delay to determine the *in vivo* internalization status of new probes, as highlighted by the pancreatic signal. These data support a hypothesis that in certain cases, PET imaging with ^140^Nd provides a localized signal only if a vector is internalized. This capability may prove useful in future drug development where *in vivo* internalization is critical for drug action.

Overall, the statistical analysis proves that the images generated using a DOTA-based ^140^Nd/^140^Pr *in vivo* generator are significantly altered from the true distribution of the tracer. With more development, it may be possible to use this technique to determine specific details of the interaction between the tracer and its molecular target. While the *p*-values are often less than the nominal 0.05 designation for statistical significance, *practical* significance is weakened by a large inter-subject variability. This means that further development in the ^140^Nd *in vivo* generator system will, for the time-being, remain an invasive procedure relegated to pre-clinical drug development.

## Conclusion

In this study, we showed that the non-internalizing tracer ^140^Nd-DOTA-LM3 accumulates in the pancreas and releases ^140^Pr^3+^ into the blood stream where it quickly redistributes to the liver and lungs. We hope that further work will lead to the development of internalization sensitive PET probes using ^140^Nd as the radiolabel. The experimental set up described here with pre- and post-mortem imaging should facilitate that development as it allows direct quantification of the parent (^140^Nd, post-mortem) and daughter (^140^Pr, pre-mortem) in the same subject. The ability to determine the tissue-dependent internalization of pharmaceuticals using PET would aid greatly in drug delivery designs where cellular internalization is crucial to drug action.

## Ethics Statement

All animal experiments were performed under a protocol approved by the National Animal Experiments Inspectorate of Denmark.

## Author Contributions

GS, LK, CN, KMJ, and UK initiated the project and conceived the experiments. UK and KJ coordinated and performed production and collections of ^140^Nd from ISOLDE-CERN. GS, JF, AJ, and AF prepared and performed the radiochemistry and quality control. LK and CN performed the *in vivo* and *ex vivo* work. GS, LK, CN, AJ, JF, HM, DJ, AK, KMJ, and KJ contributed in the interpretation of results and final design of experiments. All authors provided critical input into the final work and approve of its publication.

## Conflict of Interest Statement

The authors declare that the research was conducted in the absence of any commercial or financial relationships that could be construed as a potential conflict of interest.
