# Supplementary material for: Neodymium-140 DOTA-LM3: Evaluation of an In Vivo Generator for PET with a Non-Internalizing Vector
Source: Front Med (Lausanne). 2017 Jul 12;4:98. doi: 10.3389/fmed.2017.00098 (PMC5506079; doi:10.3389/fmed.2017.00098)
Supplement: Supplementary file 1 [file Data_Sheet_1.DOCX]

**Supplemental Materials:**

**The unit %IS/g, and Partial Volume Effects due to positron range:**

Throughout the text we have used a unit, *%IS/g*, to denote the “percent of injected annihilation signal per gram tissue”. This is in contrast to the common unit, *%ID/g* (percent of injected dose per gram), which is typically used to report relative radiotracer concentrations. We made the decision to change units for two reasons: first because the EC-delayed positron is not guaranteed to be emitted at the same location as the initial EC decay; and second, because the abnormally high energy of positrons emitted in the decay of ^140^Pr leads to large partial volume effects which alter the relationship between the decay distribution and the annihilation distribution.

We can consider the ^140^Nd (parent) spatial decay distribution, $P(r)$, the ^140^Pr (daughter) decay distribution, $D(r)$, and the annihilation distribution $H(r)$. $P(r)$ maps to $D(r)$through biological redistribution, and $D(r)$maps to $H(r)$by point spreading due to the positron range and detector resolution. The biological redistribution is the subject of the manuscript and is discussed in the text. The partial volume effect as a result of point-spreading is also very important for quantification of the PET images and is treated here:

The positron endpoint energy for ^140^Pr decay is 2.366 MeV. This is considerably higher than the positron endpoint energy for commonly used PET radioisotopes like ^18^F (the ^18^F endpoint is 0.633 MeV). The higher energy from ^140^Pr decay leads to an extended positron range, which in turn can distort PET quantification. For many isotopes this is not a critical issue because the positron range is small compared to the size regions of interest (ROIs). However, in the case of ^140^Pr the average range of the positrons is around 4-5 mm, with the highest energy positrons reaching over 11 mm. For small ROI’s (µL to mL) this either leads to an overestimation of the size of the ROI (if the ROI has been drawn based upon the PET data), or it leads to an underestimation of the amount of tracer in the ROI (if the ROI is drawn based upon anatomical imaging like CT or MRI). In either case, the PET signal in an ROI will be related to the tracer distribution in a size dependent manner, which is termed a “partial volume effect”.

It is possible to calculate the magnitude of the range-related partial volume effect as a function of ROI size. Starting with the daughter distribution, *D(r’)*, the PET the annihilation concentration, *H(r)* is given by:

$$H(r)=A \int D(r’) F(|r-r’|)dr’$$

where $F(|r-r’|)$ is the probability of an annihilation event occurring at a distance $|r-r’|$ from a source point, and *A* is simply a scaling constant. The function *F* is endpoint-energy dependent, and is equal to the positron energy distribution (i.e. the beta spectrum) mapped onto range-space through the use of electron range tables (like NIST’s ESTAR database).

The integrated PET signal, $W_{ROI}$ ,for a given ROI will be the integral of the annihilation concentration, $H(r)$*,* over the ROI volume:

$$W_{ROI}= \int_{ROI} H(r)dr$$

whereas the number of decays that occur within the ROI, $Q_{ROI}$, is

$$Q_{ROI}=\int_{ROI} D\left( r \right)dr$$

$W_{ROI}$and $Q_{ROI}$ are not equivalent since $W_{ROI}$ may include annihilation events from positrons originating outside the ROI and excludes some annihilation events from positrons originating inside of the ROI. The factor relating $W_{ROI}$ and *Q*, *S*, is given by

$$S =W_{ROI} /Q_{ROI}$$

In the special case where an ROI is homogenous, spherical, and is isolated from all other sources of positrons (*i.e.,* a xenograft tumor that is far away from tissues with high tracer accumulation) then the dependence of $S$ on the ROI volume, $V$*,* can be calculated:

$$S(V) = \frac{W_{ROI}}{\int_{\beta+range} H(r)dr}$$

This result can be validated experimentally with phantoms, which was done in the present case with a series of test-tubes, all containing an equal amount of radioactivity roughly (300kBq of ^140^Nd/^140^Pr in equilibrium), but diluted with different amounts of aqueous solution, ranging from 10 µL to 1000 µL. The tubes were scanned using the methodology described in the text for the small animal imaging.

Although the shapes of the phantom drops were not exactly spherical, the result agreed well with the calculation (figure S1). This phantom experiment is not meant to reflect a rigorous mathematical treatment of the partial volume correction. Instead intended to show that the partial volume effect can significantly alter the ROI quantification, especially for small ROI volumes. While this effect is often neglected in small animal imaging, when high energy positrons are used the correction cannot be assumed to be negligible.

Using the partial volume correction factor $S(V)$, as a function of ROI volume, the PET quantifications from the manuscript can be rescaled. The results are shown in table S1 compared to the *ex vivo* biodistribution data. As stated above, although the mathematical treatment is not rigorous, the results of the rescaling show that the biodistribution and PET data are better reconciled. A more complete approach to the partial volume correction may lead to better agreement for the PET and well-counter data.

**Figure S1:** The partial volume effect is visualized as the percentage of intra-ROI ^140^Pr decays that lead to annihilation events inside the ROI as a function of ROI volume. Or, in other words, the percentage of positrons emitted in the ROI that also annihilate in the ROI. The curve shows the spherical phantom model calculation and the points show the test-tube phantom result.

**Table S1:** A comparison of the tumor post-mortem PET signal in %IS/g with the partial volume corrected PET signal (PVC-PET) in %ID/g and the ex-vivo well counter results in %ID/g. The partial volume correction was implemented by scaling dividing the PET result by $S(V)$ from the calculation shown in Figure S1.

|  | **PET** | **PVC-PET** | **well counter** |
| --- | --- | --- | --- |
|  | %IS/g | %ID/g | %ID/g |
| **5MBq/nmol DOTA-LM3** | 0.40 ±0.15 | 3.0 ±1.0 | 6.3 ±2.3 |
| **2.5MBq/nmol DOTA-LM3** | 0.19 ±0.07 | 1.5 ±0.4 | 3.7 ±1.3 |
| **^140^Nd in saline** | 0.26 ±0.04 | 2.6 ±1.1 | 4.6 ±2.3 |
